# Risk-Prediction Model for Transfusion of Erythrocyte Concentrate During Extracorporeal Circulation in Coronary Surgery

**DOI:** 10.21470/1678-9741-2020-0322

**Published:** 2021

**Authors:** Patrícia Pinheiro Paiva, Filipe Miguel Leite, Pedro E. Antunes, Manuel J. Antunes

**Affiliations:** 1 Department of Clinical Pharmacology, Hospital and University Centre of Coimbra, Coimbra, Portugal.; 2 Department of Clinical Pharmacology, University of Coimbra Faculty of Medicine, Coimbra, Portugal.; 3 Department of Cardiothoracic Surgery, Hospital and University Centre of Coimbra, Coimbra, Portugal.; 4 Department of Cardiothoracic Surgery, University of Coimbra Faculty of Medicine, Coimbra, Portugal.

**Keywords:** Blood Transfusion, Coronary Artery Bypass, Cardiopulmonary Bypass, Risk Factors, Risk-Prediction Model

## Abstract

**Introduction:**

Our objective was to identify preoperative risk factors and to develop and validate a risk-prediction model for the need for blood (erythrocyte concentrate [EC]) transfusion during extracorporeal circulation (ECC) in patients undergoing coronary artery bypass grafting (CABG).

**Methods:**

This is a retrospective observational study including 530 consecutive patients who underwent isolated on-pump CABG at our Centre over a full two-year period. The risk model was developed and validated by logistic regression and bootstrap analysis. Discrimination and calibration were assessed using the area under the receiver operating characteristic curve (AUC) and the Hosmer-Lemeshow (H-L) test, respectively.

**Results:**

EC transfusion during ECC was required in 91 patients (17.2%). Of these, the majority were transfused with one (54.9%) or two (41.8%) EC units. The final model covariates (reported as odds ratios; 95% confidence interval) were age (1.07; 1.02-1.13), glomerular filtration rate (0.98; 0.96-1.00), body surface area (0.95; 0.92-0.98), peripheral vascular disease (3.03; 1.01-9.05), cerebrovascular disease (4.58; 1.29-16.18), and hematocrit (0.55; 0.48-0.63). The risk model developed has an excellent discriminatory power (AUC: 0,963). The results of the H-L test showed that the model predicts accurately both on average and across the ranges of deciles of risk.

**Conclusions:**

A risk-prediction model for EC transfusion during ECC was developed, which performed adequately in terms of discrimination, calibration, and stability over a wide spectrum of risk. It can be used as an instrument to provide accurate information about the need for EC transfusion during ECC, and as a valuable adjunct for local improvement of clinical practice.

**Table:** 

Abbreviations, acronyms & symbols				
**AMI**	**= Acute myocardial infarction**		**GFR**	**= Glomerular filtration rate**
**AUC**	**= Area under the ROC curve**		**H-L**	**= Hosmer-Lemeshow**
**BRiSc**	**= Papworth Bleeding Risk Score**		**Htc**	**= Hematocrit**
**CABG**	**= Coronary artery bypass grafting**		**LV**	**= Left ventricular**
**CCS**	**= Canadian Cardiovascular Society**		**OR**	**= Odds ratio**
**CI**	**= Confidence interval**		**ROC**	**= Receiver operating characteristic**
**CPB**	**= Cardiopulmonary bypass**		**SD**	**= Standard deviation**
**EC**	**= Erythrocyte concentrate**		**TEC**	**= Transfusion of erythrocyte concentrate**
**ECC**	**= Extracorporeal circulation**		**TRACK**	**= Transfusion Risk and Clinical Knowledge**
**ECu**	**= Erythrocyte concentrate units**		**TRUST**	**= Transfusion Risk Understanding Scoring Tool**
**EF**	**= Ejection fraction**			

**OR=odds ratio:**
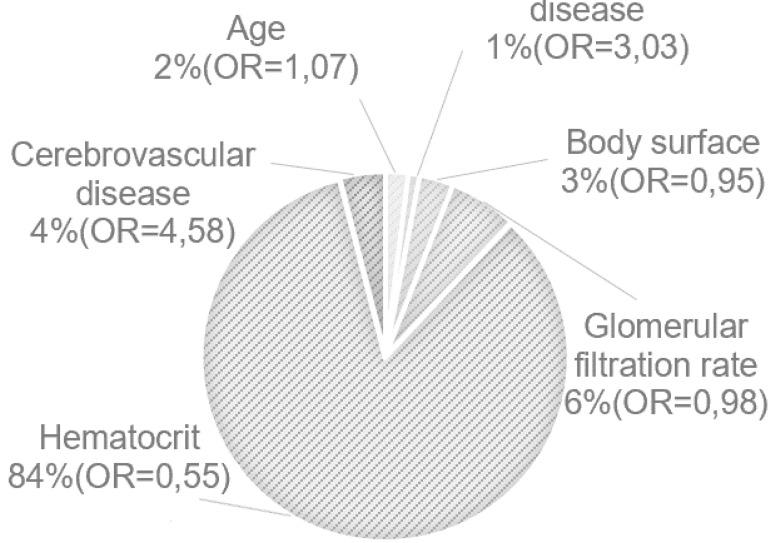
Key Question: What is the risk of the need for use of erythrocyte concentrate (EC) during cardiopulmonary bypass? Key Findings: Risk factors with the greatest prediction for EC transfusion. Take-Home Message: The implementation of this model would be an important step in optimizing and improving the quality of surgery.

## INTRODUCTION

Cardiac surgery under cardiopulmonary bypass (CPB) is associated with the transfusion of blood products^[1]^. Approximately 20% of all transfusions in the United States of America are associated with cardiac surgery^[2,3]^, and some studies report that more than half of patients undergoing cardiac surgery receive blood products during the perioperative period^[4]^. However, despite advances and efforts to conserve blood, protocols and indications for blood transfusion vary from institution to institution^[2,5,6]^. Although there is currently less need for transfusion, there are many patients who still need it and there are some variables that can predict the need for its use.

Transfusions are associated with a higher risk for the patient, as well as with a great financial cost^[7]^. Risks related to the use of blood include ABO/Rh incompatibility, sepsis, immunosuppression and viral transmission (hepatitis B, C, and human immunodeficiency virus), and association with increased morbidity and mortality^[2,5]^. There are several methods, pharmacological or non-pharmacological, that help to reduce bleeding and transfusion. The main pharmacological approaches are the use of tranexamic acid or epsilon-aminocaproic acid. Non-pharmacological methods include autologous retrograde transfusion and autotransfusion^[7]^. The use of these various methods, either alone or in combination, can reduce the number of transfusions needed^[2,7]^.

Analysis of the results is important to improve the provision of health care^[8]^. An essential part of the preoperative study of a candidate for cardiac surgery is the assessment of the need for transfusion. Risk prediction, defined as the ability to predict the outcome of the given act, can provide crucial information in several areas: for the patient and the family, who can be correctly informed about the estimated risk, increasing the accuracy of the expectation; and for the attending physician, who will be able to better assess the respective risk-benefit ratio and compare it with that of other therapeutic options^[9]^. On the other hand, it allows a constant assessment of the quality of performance of the system and, consequently, improvement in the health care provided^[10,11]^.

In this study, we intended to evaluate the influence of preoperative variables in the consumption of blood products in patients undergoing isolated coronary artery bypass grafting (CABG). The main objectives are analysis of the consumption of erythrocyte concentrate (EC) and identification of the respective risk factors and to build and validate a risk-prediction model for EC transfusion during CABG that allows the identification of risk groups within the population, which may eventually be studied in order to use blood conservation techniques, as well as the optimization of the relationship with the blood service for better organization and management of resources.

## METHODS

### Study Design and Population Selection

This is a monocentric, observational, and retrospective study consisting of 530 patients who underwent consecutive isolated CABG with CPB, at our Centre, over a full two-year period. Urgent, emergent surgery and reoperations were included in this study. Exclusion criteria were associated surgical procedures and CABG without the use of CPB (off-pump CABG). The transfusion of other blood products was not studied. All ethical issues were fulfilled in carrying out this study.

### Origin and Collection of Data

The data referring to this population were obtained by consultation of the clinical file and individual CPB data record and of the electronic system of the Clinical Pathology Department, to retrieve the preoperative serum values of hematocrit (Htc) and creatinine.

### Surgical Technique and Conduction of CPB

Priming of the extracorporeal circulation circuit was, as a general rule, performed with 1,000-1,100 cc of isotonic crystalloid solution. Whenever possible and desirable, the retrograde autologous priming technique was used for the partial or total elimination of this volume. One EC unit was usually included in the prime when the preoperative Htc was < 34%. Systemic heparinization consisted of the administration of 300 IU/kg of sodium heparin. CPB was performed with a non-pulsatile flow (roller pump) and a membrane oxygenator; the perfusion pressure was electively maintained between 55 and 60 mmHg. During CPB, a 20% mannitol solution (5 cc/kg) was routinely administered. Overall, EC transfusion was performed when the Htc value under CPB was < 24%.

Cardioplegia was not used and all distal anastomoses were performed with the heart beating or in ventricular fibrillation without aortic cross-clamping, with moderate hypothermia (32 ºC), and with a decompressed left ventricle by a cannula introduced through the right upper pulmonary vein. Proximal anastomoses were performed with the heart beating and, as a general rule, during a single period of partial clamping of the aorta. In some cases, the aorta was untouched and the anastomoses performed with one or two periods of very low flow, as described before^[12]^. At the end of the CPB, all the blood retained in the circuit was reinfused. Heparinization was reversed with administration of protamine, at a dose of 1.5 mg/kg. Blood recovery systems, such as ultrafiltration or Cell Saver, were not used.

### Statistical Analysis

Data are summarized as means and standard deviations for continuous variables and as frequencies and percentages for categorical variables. For each sample of data referring to continuous variables, the coefficients of asymmetry (skewness) and kurtosis (tailedness) were determined, as well as the respective standard errors. The sample was considered to have a normal distribution if the result of dividing the asymmetry and the kurtosis coefficients by standard errors was less than two. Student's *t*-test for two samples was performed differently, depending on whether it was assumed that the samples to be compared had or did not have different variances. The variances were previously compared using the Levene’s test.

### Risk Model - Development and Validation

The EC transfusion was dichotomized, considering the transfusion of at least one unit of EC during the CPB period as a value 1 and the opposite as value 0. The criterion for selecting the preoperative variables to be included in the analysis had considered its potential relevance in relation to the result under study and observations of previously published works.

The preoperative variables selected for analysis were age, gender, body surface, body mass index, diabetes mellitus, hypertension, recent smoking, peripheral vascular disease, cerebrovascular disease, chronic obstructive pulmonary disease, Htc, serum creatinine, glomerular filtration rate (GFR), severe angina (Canadian Cardiovascular Society class III/IV), recent acute myocardial infarction (AMI) (< 30 days), history of AMI, left ventricular (LV) dysfunction (EF < 40%), three-vessel coronary disease, non-elective surgery, and reoperation.

The entire population was used to build the risk model. This was developed using the logistic regression method associated with the random sampling technique with replacement (bootstrapping). Initially, a comparative analysis of the preoperative variables selected in the groups of patients with and without EC transfusion was performed, with the univariate analysis being performed using the Chi-square (χ^2^) test and Fisher’s test for the categorical variables, and the Student *t*-test and Mann-Whitney U test for the continuous variables. Variables that in the univariate analysis showed a *P*-value < 0.20 were submitted to a multivariate study by logistic regression using the forward stepwise method. Since the effective sample is relatively small (91 patients), a *P*-value < 0.1 was used as the criterion for retention of variables in the final model.

The technique of random sampling with replacement (bootstrapping) was used in combination with the logistic regression analysis for the process of selecting the variables to include in the final model. In this way, 200 new samples were created by this technique, all with 100% of the population (n=530), and each one of them was submitted to a multivariate analysis by logistic regression using the methodology mentioned before. Only the variables that were present in more than 50% of the samples were included in the final model, the others being excluded.

The risk model was assessed for two properties: calibration and discrimination. The calibration was evaluated by the Hosmer-Lemeshow (H-L) test, which analyzes the differences between the observed results and those predicted by the model over the risk deciles. A statistically non-significant result (*P*>0.05) suggests a good global calibration of the model. In order to provide more detailed information on the model's behavior in relation to a risk spectrum, a graph with the observed and predicted values for each of the risk decile groups was constructed.

The discriminatory power of the model was assessed by analyzing the area below the receiver operating characteristic (ROC) curve. The methodology used to calculate the area below the ROC curve and respective standard errors was the non-parametric approximation to the Wilcoxon-Mann-Whitney statistic. If the area obtained is > 0.7, it can be concluded that the model has an acceptable discriminatory power and, consequently, can be used in ordering patients in treatment groups. The R^2^ Nagelkerke value was also determined, which can give a measure of the percentage of explanation that the variables identified in the logistic regression have to predict the event under study (*e.g*., R^2^ Nagelkerke=0.521 means that 52.1% of the variation found in an event are explained by the block of variables included in the model).

The statistical analysis was performed using the IBM Corp. Released 2012, IBM SPSS Statistics for Windows, Version 21, Armonk, NY: IBM Corp.

## RESULTS

Table 1 shows the demographic and clinical characteristics of the study population. The mean age was 64.5±9.4 years and most of the patients (85.5%) were male.

**Table 1:** Patients' demographic and clinical characteristics.

Variable^a^	Population (n=530)
Age (years)	64.5±9.4
Male	453 (85.5)
Body surface area (m^2^)	1.8±0.2
Body mass index (Kg/m^2^)	27.1±2.8
Diabetes	195 (36.8)
Arterial hypertension	439 (82.8)
Recent smoking	55 (10.4)
Peripheral vascular disease	69 (13)
Cerebrovascular disease	46 (8.7)
Chronic obstructive pulmonary disease	19 (3.6)
Hematocrit (%)	41±4.4
Serum creatinine (mg/dl)	1.08±1
Glomerular filtration rate (ml/min)	82.2±27.8
Severe angina (CCS III/IV)	118 (22.3)
Recent AMI (<30 days)	108 (20.4)
History of AMI	268 (50.6)
LV dysfunction (EF <40%)	23 (4.3)
Three-vessel disease	402 (75.8)
Non-elective surgery	18 (3.4)
Reoperation	5 (0.9)

a Values are expressed as mean ± standard deviations and N (%)

AMI=acute myocardial infarction; CCS=Canadian Cardiovascular Society; EF=ejection fraction; LV=left ventricular

Table 2 describes the data related to EC transfusion in the study population. In total, the average number of EC units administered was 0.3. Ninety-one patients (17.2%) were transfused: 50 (54.9%), 38 (41.8%), and three (3.3%) patients received one, two, and three units, respectively. A total of 135 EC units (mean: 1.5 per patient) were transfused; of these, 63% were used in 41 patients (45.1%).

**Table 2:** Data related to erythrocyte concentrate transfusion in the study population.

	Nº of patients	% of total	% relative	ECu (mean ± SD)
Total population	530			0.3±0.6
Transfused population	91	17.2		1.5±0.6
Priming only	14	2.7	15.4	1.4±0.5
CPB only	61	11.5	67	1.3±0.5
Priming and CPB	16	3	17.6	2.2±0.4

CPB=cardiopulmonary bypass; ECu=erythrocyte concentrate units; SD=standard deviation

Table 3 shows the data of preoperative variables in the groups of patients with and without EC transfusion during CPB. When compared to the one that was not transfused, the population with EC transfusion was older (*P*<0.001), had smaller body surface area and body mass index, lower Htc (*P*≤0.001), worse renal function (higher creatinine levels and lower GFR; *P*<0.005), and a significantly higher prevalence of women, arterial hypertension, recent AMI, peripheral vascular disease, cerebrovascular disease, and severe angina.

**Table 3:** Univariate analysis of preoperative variables in groups of patients with (n=91) and without (n = 439) TEC during CPB.

Variable^a^	TEC (n=91)	Without TEC (n=439)	*P*-value
Age (years)	70.4±8.7	63.4±9.1	< 0.001
Women	48.1	11.9	< 0.001
Body surface area (m^2^)	1.7±0.1	1.8±0.2	< 0.001
Body mass index (Kg/m^2^)	26.2±4.3	27.8±3.8	0.001
Diabetes mellitus	42.9	35.5	0.187
Arterial hypertension	93.4	80,6	0.003
Recent smoking	31.9	51.7	0.001
Peripheral vascular disease	25.3	10.5	< 0.001
Cerebrovascular disease	16.5	7.1	0.004
Chronic obstructive pulmonary disease	3.3	3.6	0.871
Hematocrit (%)	34.9±3.4	42.2±3.4	< 0.001
Serum creatinine (mg/dl)	1.56±0.16	0.88±0.04	0.034
Glomerular filtration rate (ml/min)	61.3±21.4	86.6±27	< 0.001
Severe angina (CCS III/IV)	30.8	20.5	0.032
Recent AMI (< 30 days)	31.9	18	0.003
History of AMI	59.3	48.7	0.066
LV dysfunction (EF <40%)	4.4	4.3	0.578
Three-vessel disease	79.1	75.2	0.823
Non-elective surgery	5.5	3	0.225
Reoperation	1.1	0.9	0.866

a In cases where the unit of measurement is not refereed, the values are expressed as percentage (%).

AMI=acute myocardial infarction; CCS=Canadian Cardiovascular Society; CPB=cardiopulmonary bypass; EF=ejection fraction; LV=left ventricular; TEC=transfusion of erythrocyte concentrate

The model for predicting the risk of EC transfusion included the following variables: age, body surface, peripheral vascular disease, cerebrovascular disease, Htc, and GFR (Table 4).

**Table 4:** Risk-prediction model.

Variables	Coefficient	*P*-value	Frequency bootstrap	OR	95% CI (OR)	
Age (years)*	0.071	0.006	55.3%	1.07	1.02	1.13
Body surface area (m^2^)*	-0.052	< 0.001	95%	0.95	0.92	0.98
Peripheral vascular disease	1.107	0.048	50.5%	3.03	1.01	9.05
Cerebrovascular disease	1.521	0.018	54.2%	4.58	1.29	16.18
Hematocrit (%)*	-0.599	< 0.001	100%	0.55	0.48	0.63
Glomerular filtration rate (ml/min)*	-0.021	0.011	96.5%	0.98	0.96	1.00
Constant	27.005	< 0.001				

CI=confidence interval; OR=odds ratio

* By one increment unit.

Model: χ^2^ [6 g.l.] = 297,47, *P*<0.001.

**Fig. 1:**
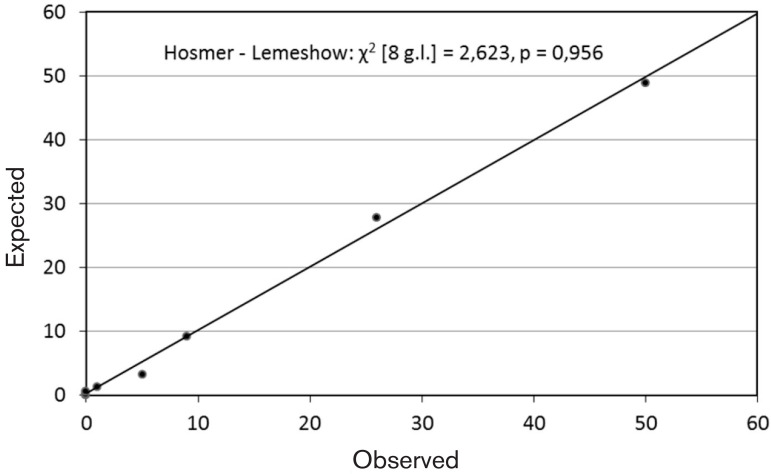
Risk-prediction model. Observed vs. expected values of risk deciles.

The model accurately predicts the risk of EC transfusion (χ^2^ [6 g. L.] = 297.47, *P*<0.001). The result of the H-L test revealed that there was no significant difference between the values observed and those predicted by the model (*P*=0.956). On the other hand, there is also a good adjustment between the observed and expected values throughout the partial analysis of the risk decision groups (Figure 1).

**Fig. 2:**
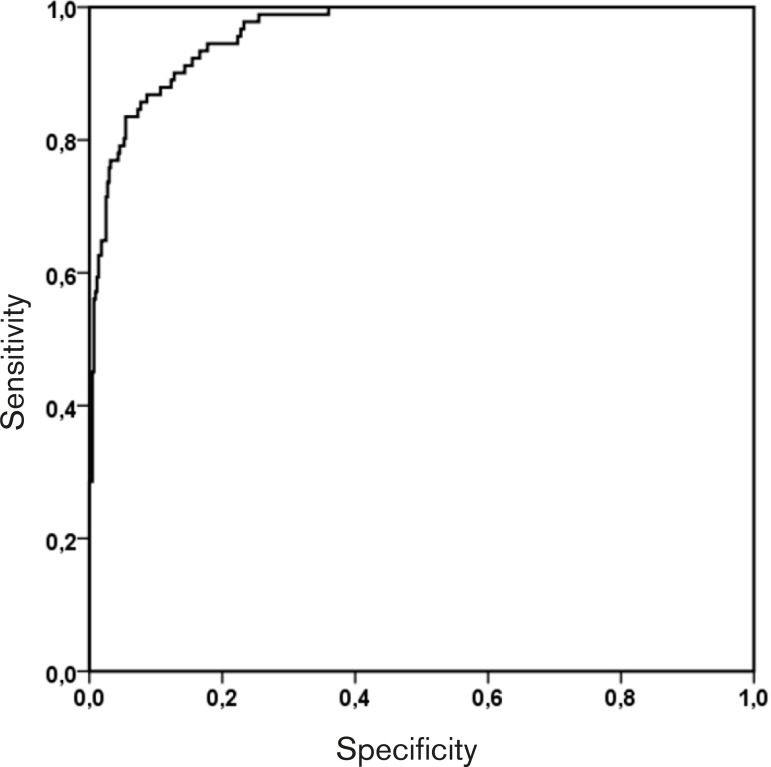
Receiver operating characteristic (ROC) curve of the risk-prediction model. The area under the model's ROC curve was 0.963 (95% confidence interval: 0.947-0.979).

About 71.5% (R^2^ Nagelkerke=0.715) of the variation found in EC transfusion during CPB is explained by the model. The relative contribution of each factor to the risk-prediction model is shown in the Visual Abstract. It should be noted that the Htc alone explains 60.2% (R^2^ Nagelkerke=0.602) of the variability found for calculating the risk predicted by the model, and, consequently, 84% is the relative contribution of this variable to the risk estimate. The ROC curve of the risk-prediction model for EC transfusion is shown in Figure 2.

The model was validated internally, obtaining for the 200 new samples tested an average value of 0.962 (95% confidence interval: 0.945-0.980) of the area below the ROC curve.

## DISCUSSION

A risk prediction model for EC transfusion was developed, which proved to be a good instrument to provide an objective individual estimate of the need for EC transfusion during CPB in our patient population. This measure can improve clinical practice in the institution, essentially regarding the allocation of available resources, decision making, informed consent, and quality control.

The results described before cannot be compared with others, since, to our knowledge, there is no record of the consumption of blood products during the CPB period itself. In general, the series published refer to the global perioperative period or the period of surgery and intensive care unit.

Once a patient becomes a candidate for cardiac surgery, an important part of the preoperative study is the assessment of the need for and risk of transfusion of blood components. Recognition of this concept is well expressed in the guidelines on patient blood management for adult cardiac surgery of the Society of Thoracic Surgeons/Society of Cardiovascular Anesthesiologists^[13]^, and of the European Association for Cardio-Thoracic Surgery/European Association of Cardiothoracic Anesthesiology^[14]^, and the guidelines on perioperative medication in adult cardiac surgery and patient blood management^[15]^.

The initial studies, on which most of the early recommendations were based, were retrospective and reinforced the idea of EC administration as a factor of poor prognosis. Classically, they compared patients who received EC *versus* patients who did not. Even though they recognized that the first group of patients were at higher risk, trying to compensate for this fact by statistical methods, the need for transfusion remained an independent marker of poor results^[16]^, an idea that remains rooted to this day^[17-19]^. However, more recent, non-retrospective studies, based on randomized trials and meta-analyses, question previous evidence.

Thus, and although there are some risk scores, such as the Transfusion Risk and Clinical Knowledge (TRACK) score^[17]^, the Transfusion Risk Understanding Scoring Tool (TRUST)^[18]^ and the Papworth Bleeding Risk Score (BRiSc)^[19]^, these did not serve as a reference. TRACK was not used because it is a score that is based on a specific population (Jehovah's Witnesses); TRUST is a score that includes all surgical procedures that require CPB; and in BRiSc, there was only one center, admitting that there are differences when the results are compared with those of the other centers.

A fundamental question raised by many of previous works is whether EC administration is a marker of poor results, so a restrictive administration policy (Htc < 24%) would lead to better results compared to a liberal administration policy (Htc < 30%). However, these same trials have shown that liberal strategies are not inferior, with lower long-term mortality, raising even the possibility of very restrictive policies to increase global mortality^[16]^.

The use of blood in coronary surgery with CPB is highly variable. In the studies published by Takai^[20]^ and Scott et al., the average number of transfused EC units was 0.9 and 2.4, respectively. Another study, involving a population of 732 patients operated on at our Centre, in the 2002-2003 biennium, reported an average value of 0.2 EC units^[21]^, which reveals instinctual stability of this indicator in quite separate temporal analyses.

The developed risk model proved to be valid, with good calibration and good discriminatory power, considering that 71.5% of the variation explained by the model is unusually high. When considering the initial variables and the values obtained by univariate analysis, it is interesting to consider that the classic *locus* of mortality in cardiac surgery from diabetes mellitus, arterial hypertension, smoking, and LV dysfunction are not risk factors. Usually, these are indicative of patients with worse general condition, with more complex and time-consuming surgeries.

In the present study, after univariate and multivariate analysis, age, body surface, peripheral vascular disease, cerebrovascular disease, Htc, and GFR were identified as the risk factors with the greatest prediction for EC transfusion. Htc represents 84% of the variation explained by the model, however the importance of the remaining variables is not negligible. Among the factors responsible for the variation of the Htc, is not only the hemorrhage, but preoperative anemia and intraoperative haemodilution are as/more significant^[16]^. Some authors argue that the body surface provides the best approximation for the total blood volume^[22]^. Having said this, and taking into account the constant volume corresponding to the volume administered in CPB, haemodilution is greater as the smaller the body surface is^[23]^.

The approach to patients with indication for CABG and who have concomitant severe carotid artery disease is controversial but falls outside the scope of this study. In these cases, the increased need for EC transfusion leads to ponder the effect of changes in carotid and cerebral circulation and in peripheral and central baroreceptors and chemoreceptors.

The association between renal dysfunction and haemodilution is well established, as well as its relationship with heart failure and the adverse events that result from it^[24]^. This baseline haemodilution in patients with renal dysfunction, associated with haemodilution inherent to CPB, is an important factor in decreasing Htc and increase the need for transfusion. Interestingly, for reasons not yet fully defined/identified, the decrease in Htc is a risk factor for renal failure^[25]^.

Hence, the implementation of this risk model can be of great importance. Preoperatively, in addition to a correct study and optimization of the patient in preparation for surgery, renal function and Htc value are particularly important, since they are the two most directly modifiable variables. At the same time, the use of this model allows the creation of a risk profile for EC administration in our patient population. Additionally, it can be used as a valuable adjunct to the improvement of clinical practice in the population treated at the institution, essentially regarding allocation of available resources, decision-making, informed consent, and quality control. The high cost of EC and other blood products, the scarcity of donors and the risks inherent to the transfusion itself implicate that administration of these products needs to be carefully evaluated.

In the extreme, this risk stratification allows modifying the surgical technique, namely through the use of surgery without CPB, or the adoption of forms of blood conservation such as the Cell Saver. Some blood conservation measures are already routinely implemented: retrograde autologous priming (whenever possible) and reinfusion of residual blood from the circuit at the end of the perfusion/intervention (still in the operating room). The CPB circuit must be prepared so that filling can be done with the smallest volume possible using tubes, circuits, reservoirs, and oxygenators with reduced volumes. Also, in our Centre, the blood drained from mediastinum and pericardium during the first hours in the intensive care is reinfused directly or after washing and filtering.

In short, the implementation of this model would be an important step in optimizing and improving the quality of surgery. Additionally, it can be used as a valuable adjunct to the improvement of clinical practice at the institution, essentially regarding the allocation of available resources, decision-making, informed consent, and quality control.

### Limitations

The limitations of this study are inherent to its retrospective design, which means that the associations found may not necessarily have a causal link. On the other hand, the calculated odds-ratios represent only an approximation of the real relative risk, which can only be calculated with prospective methodologies.

## CONCLUSION

We developed a risk-prediction model for EC transfusion that reveals an adequate performance in relation to three aspects: discrimination, calibration, and stability over a wide spectrum. Consequently, the developed risk model can be used as an instrument to provide an objective individual estimate of the need for EC transfusion during CPB in our patient population.

**Table:** 

Authors' roles & responsibilities	
PPP	Substantial contributions to the conception or design of the work; or the acquisition, analysis, or interpretation of data for the work; drafting the work or revising it critically for important intellectual content; agreement to be accountable for all aspects of the work in ensuring that questions related to the accuracy or integrity of any part of the work are appropriately investigated and resolved; final approval of the version to be published
FML	Substantial contributions to the conception or design of the work; or the acquisition, analysis, or interpretation of data for the work; drafting the work or revising it critically for important intellectual content; agreement to be accountable for all aspects of the work in ensuring that questions related to the accuracy or integrity of any part of the work are appropriately investigated and resolved; final approval of the version to be published
PEA	Substantial contributions to the conception or design of the work; or the acquisition, analysis, or interpretation of data for the work; drafting the work or revising it critically for important intellectual content; agreement to be accountable for all aspects of the work in ensuring that questions related to the accuracy or integrity of any part of the work are appropriately investigated and resolved; final approval of the version to be published
MJA	Substantial contributions to the conception or design of the work; or the acquisition, analysis, or interpretation of data for the work; drafting the work or revising it critically for important intellectual content; agreement to be accountable for all aspects of the work in ensuring that questions related to the accuracy or integrity of any part of the work are appropriately investigated and resolved; final approval of the version to be published
